# CMakeCatchTemplate: A C++ template project

**DOI:** 10.5334/jors.319

**Published:** 2021-07-16

**Authors:** Thomas Dowrick, Mian Ahmed, Stephen Thompson, James Hetherington, Jonathan Cooper, Matt Clarkson

**Affiliations:** Wellcome EPSRC Centre for Interventional and Surgical Sciences, UCL; Wellcome EPSRC Centre for Interventional and Surgical Sciences, UCL; Wellcome EPSRC Centre for Interventional and Surgical Sciences, UCL; Centre for Advanced Research Computing, UCL; Research Software Development Group, Research IT Services, UCL; Wellcome EPSRC Centre for Interventional and Surgical Sciences, UCL

**Keywords:** CMake, C++, Project template, Scaffolding

## Abstract

CMakeCatchTemplate (https://github.com/MattClarkson/CMakeCatchTemplate) is a project to provide a starting structure for C++ projects configured with CMake, that can be customised to work in a variety of scenarios, allowing developers to deploy new algorithms to users in a shorter timeframe. Main features include a SuperBuild to build optional dependencies; unit tests using Catch; support for CUDA, OpenMP and MPI; examples of command line and GUI applications; Doxygen integration; Continuous Integration templates and support for building/deploying Python modules.

## Introduction

CMakeCatchTemplate was originally developed as a teaching aid for UCL’s Research Computing with C++ course, to demonstrate how a complex C++ project might be structured, and provide a starting point for student’s own projects. Subsequently, the project has been expanded upon and is now employed as a template project for a range of C++ projects within the Wellcome/EPSRC Centre for Interventional and Surgical Sciences (WEISS),^1–2^ as part of the SNAPPY library.^3^ By reducing the amount of boilerplate code and providing support for a range of common libraries for scientific computing, the template allows a developer to quickly prototype and release a new library.

In addition to scaffolding to provide the structure for a C++ project, particular features provided are:

1. A Meta-Build, also known as a SuperBuild, to optionally download and build any of the following: Boost, Eigen, FLANN, OpenCV, glog, gflags, VTK, PCL and ArrayFire. This results in a top-level build folder containing the compiled dependencies, and then a sub-folder containing the compiled code of this project.
2. A single library into which the user can code their main algorithms.
3. Unit tests using Catch.
4. A single command line application, to give the end user a minimalist runnable program.
5. Basic examples of how to create a Qt+VTK, Qt+OpenGL or QML+VTK user interface, ensuring the VTK render engine works in Qt or QML framework, on Windows, Linux and Mac. Qt installation is not included, and must be carried out separately.
6. CPack setup to produce installers for the GUI apps.
7. KWStyle config, to check for consistent code style (requires KWStyle installed on user’s system).
8. CppCheck config, to check for performance, style and correctness issues (requires CppCheck installed on user’s system).
9. Doxygen config, so you can generate documentation via make docs, or a DOCS task in Visual Studio.
10. GitHub CI, Travis and Appveyor examples, to register the code with a Continuous Integration service.
11. An example of the CMake required to build Python interfaces to your C++ code, using Boost.Python.
12. An example of the CMake required to build Python interfaces to your C++ code, using pybind11.^4^ Also includes an example of passing numpy/OpenCV data through Boost.Python, thanks to Gregory Kramida’s pyboostcvconverter.^5^
13. An example of the CMake required to export a C-style module into Unity.
14. Support for OpenMP, which is passed through to FLANN and OpenCV.
15. Support for CUDA, which is passed through to FLANN, OpenCV and PCL.
16. Support for MPI, which by default sets up the C++ libraries.
17. Support for Python Wheels, thanks to Matthew Brett’s multibuild.^6^

In practice, most developers will only require a subset of the available features for a particular project. We envisage a range of project types that might commonly be implemented:

- C++ library, with C++ command line interfaces.
- C++ library with C++ user interface, using Qt or QML.
- C++ library deployed as a Python module to PyPI, with separate Python code written outside of the project to make use of the module.
- Arrayfire/CUDA project.
- C++ library used in an environment such as Unity, which is developed outside of the project.

The template is a starting point for developers wishing to avoid some of the overheads associated with setting up a new project, but still requires input from the user to customise to their particular application, and a working knowledge of CMake to get the maximum benefit.

In addition to the combination of functionality listed above, which are not found in any single existing C++ project template,^7^ particular features which differentiate CMakeCatchTemplate include the ability to generate Python wheels, support for CUDA/OpenMP/MPI, and examples usage for Qt/QML applications. Further, the laborious process of manually renaming/editing files upon first creating a project, as is typically required when using other templates, is removed by the addition of the *CMakeCatchTemplateRenamer* ^8^ helper script.

## 2 Implementation and Architecture

Features have been added over time based on feedback/requests from users, who are typically C++ developers aiming to write a small algorithm library for a particular application and get it into the hands of their users, who may not themselves be familiar with C++.

### 2.1 Project Structure

**Figure 1:**
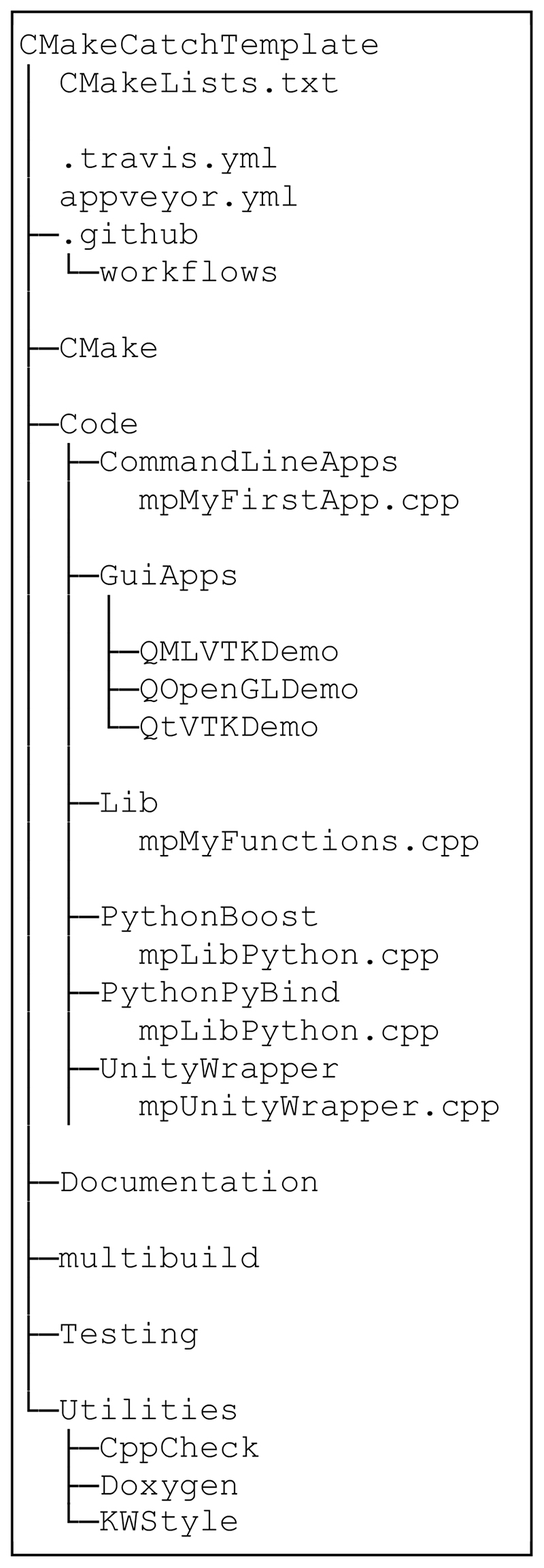
Simplified file structure showing key project components.

**Figure 2:**
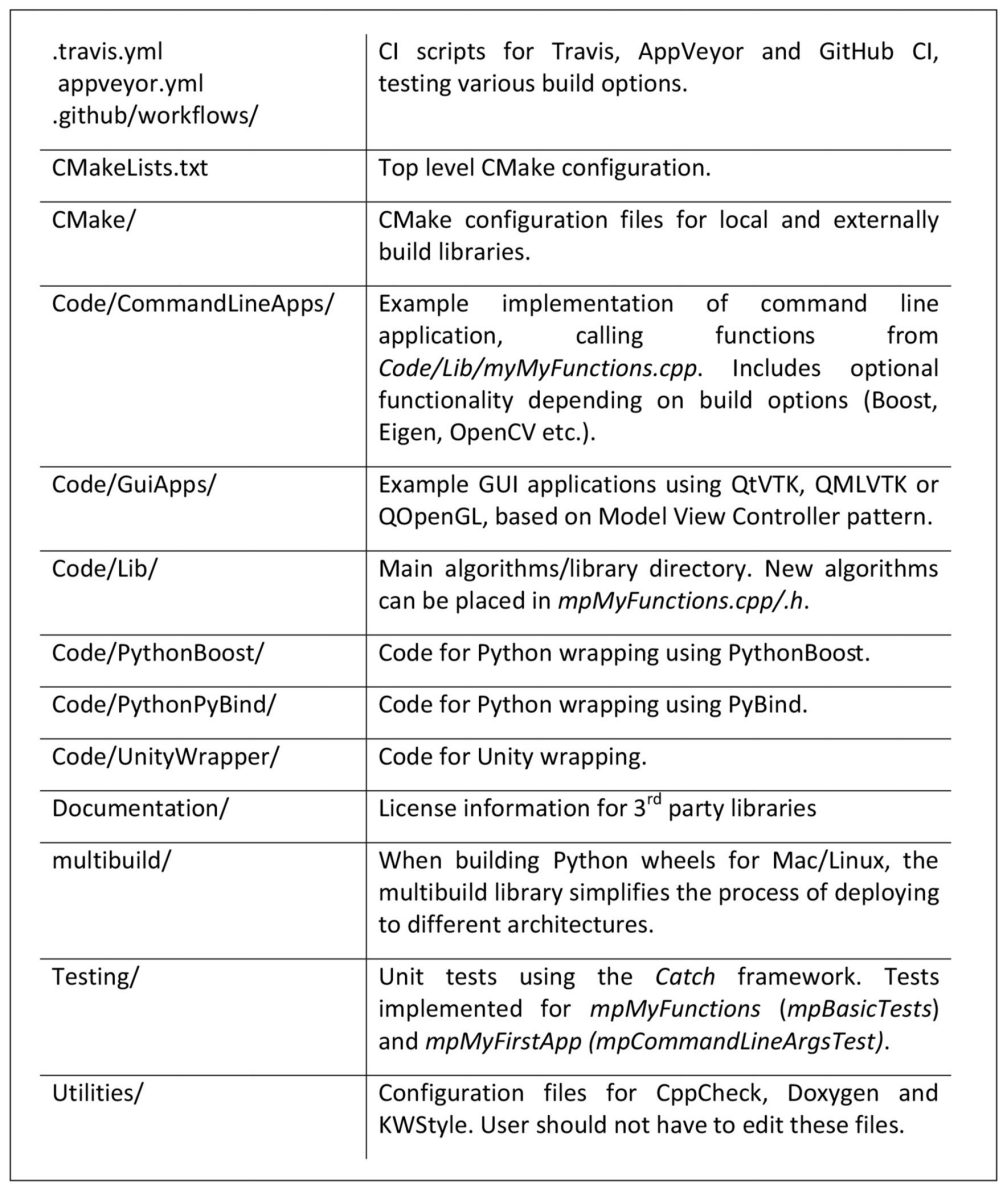
Explanation of key directories/files.

The template provides a ready-made structure for C++ projects (Figures 1 and 2). The user can build on this structure by adding their own code, primarily in *Code/Lib*.

### 2.2 Build Options

**Figure 3:**
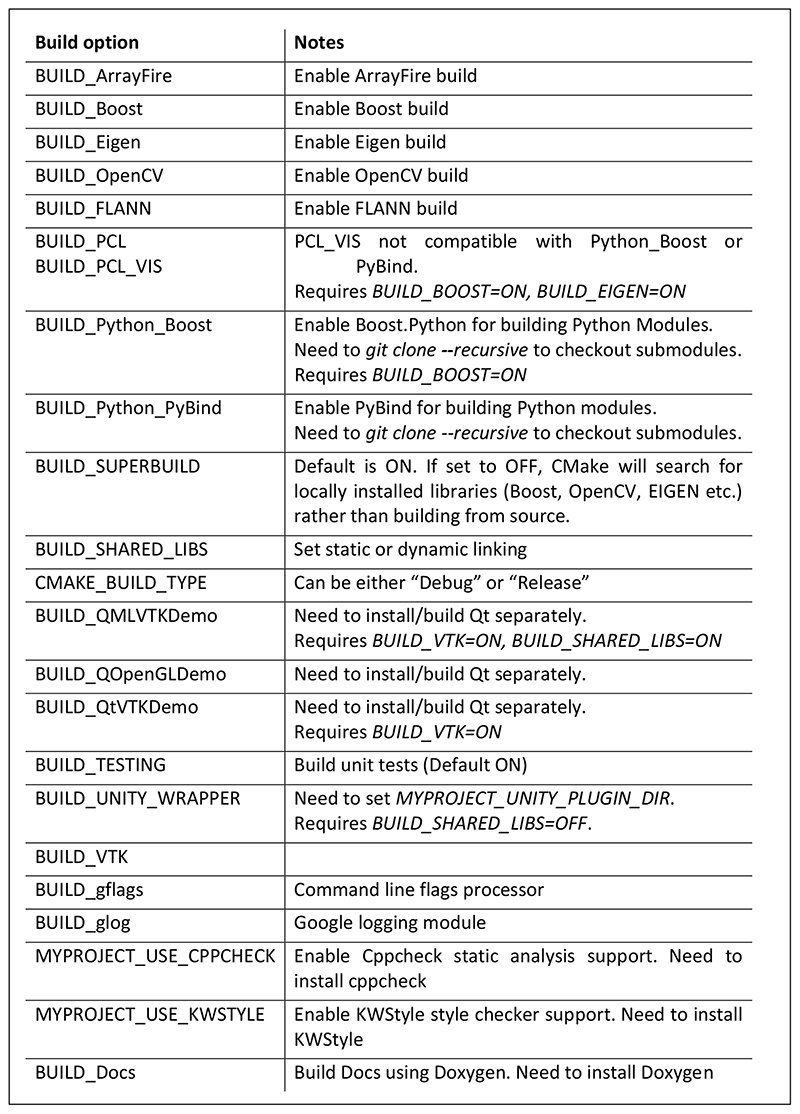
Build options.

Build options (Figure 3) can be passed to CMake depending on the external libraries required.

### 2.3 Python Wheels

Deploying cross-platform Python wheels from C++ code can be a difficult process. To help, we have been inspired and assisted by Matthew Brett’s multibuild,^9^ with the included *travis.yml* and *appveyor.yml* used to deploy the example code included with CMakeCatchTemplate to PyPI.^9^ Users can re-use, or modify these configurations as needed.

### 2.4 Further Customisation

We believe that it is preferable to provide tested code that may not be needed by all users, rather than requiring the user to spend time writing potentially complex CMake. However, once a user has established a working build for their particular needs, it is possible to pare down the codebase by removing segments that will no longer be needed.

For example:

- In the *Code* directory, subfolders for GUIs or Python/Unity bindings can be deleted if not required.
- 3^rd^ party libraries can be removed from the top level *CMakeLists.txt* e.g. by removing references to *mpAddBoost* or *mpIncludeBoost*.
- Unused items from the *Utilities* folder can be removed.
- CI files (*.travis.yml, appveyor.yml, .github/workflows*) can be modified/removed.

### 2.5 Quality Control

Continous Integration (CI) is used to test a range of CMake build options, and provide example use cases.

GitHub workflows are used to build/test some common configurations (Boost, OpenCV, PyBoost, PyBind, Qt) on an Ubuntu VM, which also provide a starting point for users to build their own projects. Workflows are stored in the *.github/workflows* directory.

A more complex example, of building a project and deploying to PyPI, is tested using Travis (Mac/Linux) and AppVeyor (Windows). Users can find more details in the *.travis.yml* and *appveyor.yml* files in the top level directory.

Further examples can be found in projects which have been derived from CMakeCatchTemplate – *scikitsurgery-opencvcpp*[^10^ and *scikit-surgerypclcpp*,^11^ which each implement new algorithms within the /*Code/Lib* folders, and unit tests in /*Testing*. Again, the Travis and AppVeyor configuration files detail an up-to-date build procedure.

The default project structure includes some rudimentary unit tests. Once built, these tests are placed in the *MYPROJECT-build/bin* folder. The CI examples demonstrate how to run tests. The included tests serve to illustrate the structure/framework that has been implemented. Further unit tests should be added by the user once they have added their own code to the project.

## 3 Availability

### Operating System

Tested locally on:

Windows - Windows 8/10, VS2013, CMake 3.6.3, Qt 5.4.2

Linux - Centos 7/Ubuntu 16, g++ 4.8.5, CMake 3.5.1, Qt 5.6.2

Mac - OSX 10.10.5, clang 6.0, CMake 3.9.4, Qt 5.6.2

Refer to CI files for details on other test environments used.

### Programming Language

C++ 11

### Additional System Requirements

None

### Dependencies

CMake > 3.5

If Qt is enabled, minimum version is Qt5.

### Software Location

#### Archive

      ***Name:*** CMakeCatchTemplate

      ***Persistent identifier:**https://doi.org/10.5281/zenodo.4954783*

      ***Licence:** BSD 3-clause*

      ***Publisher:** Zenodo*

      ***Version published:** 0.3*

      ***Date published:** 15/06/2021*

#### Code repository

      ***Name:*** CMakeCatchTemplate

      ***Persistent identifier:**https://github.com/MattClarkson/CMakeCatchTemplate*

      ***Licence:** BSD 3-clause*

      ***Date published:** 16/01/2020*

      ***Code repository:*** GitHub

### Language

C++/CMake

## 4 Reuse Potential

This software can be used for rapid prototyping and deployment of novel C++ algorithms amongst research users, and also for teaching environments/student projects where CMake development is beyond the scope of the course material.

While long term support is not explicitly guaranteed for this project, the authors continue to maintain the repository and will reply to any GitHub issues raised, and this is not expected to change. Further to this, the existing CI infrastructure will indicate if the software is still working as expected.

Users wishing to contribute towards the software could include additional libraries, develop new build/CI scripts for different applications, or share projects that they have made using the template.

## NOTES

1 https://github.com/UCL/scikit-surgeryopencvcpp.

2 https://github.com/UCL/scikit-surgerypclcpp.

3 https://weisslab.cs.ucl.ac.uk/WEISS/. PlatformManagement/SNAPPY/wikis/home.

4 https://github.com/pybind/cmake_example.

5 https://github.com/Algomorph/pyboostcvconverter.

6 https://github.com/matthew-brett/multibuild.

7 https://github.com/TheLartians/ModernCppStarter. https://github.com/cginternals/cmake-init. https://github.com/Lectem/cpp-boilerplate. https://github.com/arnavb/cpp14-project-template. https://github.com/joshpeterson/cpp-template.

8 https://github.com/MattClarkson/CMakeTemplateRenamer.

9 https://pypi.org/project/CMakeCatchTemplate/.

10 https://github.com/UCL/scikit-surgeryopencvcpp.

11 https://github.com/UCL/scikit-surgerypclcpp.

